# The association between serum magnesium levels and community-acquired pneumonia 30-day mortality

**DOI:** 10.1186/s12879-018-3627-2

**Published:** 2018-12-27

**Authors:** Roni Nasser, Mohammad E. Naffaa, Tanya Mashiach, Zaher S. Azzam, Eyal Braun

**Affiliations:** 1 Department of Internal Medicine “B”, Ramabm Health Care Campus, HaAliya HaShniya St 8, 3109601 Haifa, Israel; 2 Rheumatology unit, Galilee Medical Center, Nahariya, Israel; 3 Epidemiology and Biostatistics Unit, Rambam Health Care Campus, Haifa, Israel; 4 The Rappaport’s Faculty of Medicine, The Technion Institute, Haifa, Israel; 5 Department of Internal Medicine “H”, Ramabm Health Care Campus, Haifa, Israel

**Keywords:** Pneumonia, Hypomagnesemia, Hypermagnesemia, Mortality

## Abstract

**Background:**

Community acquired pneumonia (CAP) is a common illness affecting hundreds of millions worldwide. Few studies have investigated the relationship between serum magnesium levels and outcomes of these patients. We aimed to study the association between serum magnesium levels and 30-day mortality among patients with CAP.

**Methods:**

Retrospective overview of patients hospitalized with CAP between January 1, 2010 and December 31, 2016. Participants were analyzed retrospectively in order to identify the risk factors for a primary endpoint of 30-day mortality. Normal levels of magnesium levels in our laboratory varies between 1.35 and 2.4 mg/dl.

**Results:**

3851 patients were included in our cohort. Age > 75 years, blood urea nitrogen (BUN) > 20 mg/dl, hypoalbuminemia, and abnormal levels of magnesium were all associated with increased risk of 30-day mortality. Normal magnesium levels were associated with the lowest mortality rate (14.7%). Notably, within the normal levels, high normal magnesium levels (2–2.4 mg/dl) were correlated with higher mortality rates (30.3%) as compared to levels that ranged between 1.35–2 mg/dl (12.9%). Hypomagnesemia and hypermagnesemia were both associated with excess of 30-day mortality, 18.4 and 50%, respectively.

**Conclusion:**

Hypomagnesemia and hypermagnesemia on admission were associated with an increased rate of 30-day mortality among adult patients hospitalized with CAP. Interestingly, magnesium levels within the upper normal limits were associated with higher mortality.

## Background

Community acquired pneumonia (CAP) is a common illness affecting hundreds of millions worldwide, with increasing hospital admissions throughout the years mainly due to the aging population. It is a major cause of mortality and morbidity in all age groups, especially elderly, despite the effectiveness of the diverse antibiotic treatment [1–3].

The prognostic scores used in the clinical settings, such as CURB 65 and Pneumonia Severity Index (PSI) are acknowledged as tools to estimate the mortality rates and thus determine the treatment setting, either outpatient or inpatient [2, 4].

Magnesium (Mg) is the second most profound intracellular mineral in the human body. It is essential for energy production, mainly by binding ATP; synthesis of DNA, RNA and proteins. Mg also plays a role in the active transport of calcium and potassium ions across cell membranes, and thus has an essential role for maintaining proper function of the neuromuscular and cardiovascular systems [5].

Magnesium deficiency has been associated with a number of clinical manifestations such as arrhythmias, cardiac insufficiency, sudden death, muscle weakness, bronchospasm, tetany, seizures, as well as hypokalemia, hypocalcaemia, hyponatremia, and hypophosphatemia [6, 7]. Several studies demonstrated that hypomagnesemia at admission or during ICU stay was associated with guarded prognosis [8, 9], and magnesium supplementation was associated with a lower mortality rate [8].

Hypermagnesemia, which is less frequent than hypomagnesemia, commonly occurs due to excessive administration of magnesium salts or magnesium-containing drugs, especially in patients with reduced renal function. It may be caused, also, by rapid mobilization from soft tissues in patients with sepsis or trauma, adrenal insufficiency and hypothyroidism. Hypermagnesemia may cause severe symptomatic hypotension, bradycardia and ECG changes like wide QRS [5, 10]. Hypermagnesemia was associated with highest rates of death in critically ill patients [11].

We recently showed that hyperphosphatemia and hypophosphatemia were independently associated with increased 30-days mortality rates in patients with CAP [12].

In this study, we aimed to examine the association between serum magnesium levels on admission and the 30-day mortality in patients with CAP.

## Methods

### Study design

Retrospective overview of patients who were admitted to Rambam Health Care Campus (RHCC), Haifa, Israel, between January 2010 and 31 December 2016. RHCC is a 1000 bed teaching hospital. The patient population is diverse, as RHCC is the major tertiary medical center for all of Northern Israel, serving more than two million residents. According to hospital records, there are 140,000 emergency department visits every year and about 90,000 inpatient admissions. The Rambam Hospital Institutional Review Board approved the study. The approval number is 0597–16-RMB. The need for informed consent was waived because of the retrospective, medical record-based design of the study. The study population included patients 18 years or older with CAP. The diagnosis of pneumonia was based upon the primary diagnosis of pneumonia on the discharge report, within the first twenty-four hours from admission. Exclusion criteria included patients younger than 18 years, those who were transferred from another acute care facilities, hospitalization during the month prior to admission, hospital-acquired pneumonia (HAP) or partial antibiotic treatment.

Patients’ data was retrieved and analyzed using Prometheus, RHCC integrated electronic medical records system. The 30-day mortality data were retrieved from Prometheus and the ministry of health. The retrieved data included age, gender; vital signs including blood pressure (BP), systolic and diastolic, heart rate (HR), Oxygen saturation (SO2) respiratory rate (RR), temperature; Comorbidities: history of prior or current malignancies (solid or hematologic), lung disease, smoking status, cardiovascular diseases, kidney diseases, immune deficiency conditions, HIV status, diabetes mellitus, liver cirrhosis, prior neurologic damage, alcohol abuse, intravenous drug abuse and nursing house residence smoking history. The Charlson’s comorbidity score was calculated based on data collected; laboratory values (first values within 48 h): Hemoglobin (Hb), White blood cell count (WBC), red blood cell distribution width (RDW), pH, partial pressure of carbon dioxide (pCO2), Serum glucose, serum creatinine, sodium, calcium, phosphorus, magnesium, blood urea nitrogen (BUN), and serum albumin.

Hematological values were measured using the Advia 120 Hematology Analyzer (Siemens Healthcare Diagnostics Deerfield, Illinois, USA). Serum glucose, serum creatinine, sodium, calcium, phosphorus, magnesium, blood urea nitrogen (BUN), and serum albumin were measured on admission Using “Dimension” (Siemens Healthcare Diagnostics Deerfield, Illinois, USA). PH, bicarbonate, partial pressure of CO2 and lactate were measured using GEM premier 3500.

The normal serum magnesium range in our laboratory is 1.35–2.4 mg/dl.

### Statistical analysis

Statistical analysis was performed by using the SPSS statistical package (SPSS, Inc., Chicago, USA) version 21.0.

Quantitative variables are expressed as mean ± SD. Qualitative variables are expressed as values and percentages. The odds ratio (OR) with 95% confidence interval (CI) was computed using bivariate logistic regression analysis. The correlation between patients’ characteristics and 30-day mortality were evaluated by *P* values derived from bivariate analysis. Multivariate forward stepwise logistic regression was used to evaluate the relation between patients’ features, co-morbidities, laboratory parameters, and 30-day mortality.

Parameters with notable level of significance (*P* < 0.1) of the bivariate association with 30-day mortality were chosen for the multivariate analysis. Bootstrap multivariate analysis was used to evaluate the accuracy of the parameters in the model by estimating standard error, confidence intervals, and bias. The area under curve (AUC) was applied in the model to assess the prognostic value of magnesium. *P* values equals or less than 0.05 was acknowledged as statistically significant.

## Results

Between January 1, 2010 and December 31, 2016, 4708 patients were diagnosed with CAP at discharge. 3851 patient had magnesium levels within 48 h. Fifty five percent were males. Median age was 72 years old. The 30-day mortality was 15.2% (587 patients) and almost the same each year during the study.

Hypomagnesemia (≤1.35 mg/dl) was detected in 240 patients (6%) and hypermagnesemia (≥2.4 mg/dl) in 26 patients (1%), while 3581 patients (93%) were normomagnesemic (1.35–2.4 mg/dl).

Table 1 shows bivariate analysis of the association between patients’ characteristics, laboratory parameters and 30-day mortality.

**Table 1:** Bivariate analysis of parameters associated with 30 days mortality

		Total		30 day mortality				95%CI	
		Number	Percent (%)	Number	Percent %	P value	OR	lower	Upper
	Total patients	3851		587	15.2%				
Age (years)	< 65	1215	32%	81	6.7%	0.000	1.00	0.00	0.00
	65–69	354	9%	31	8.8%	0.180	1.34	0.87	2.07
	70–79	947	25%	156	16.5%	0.000	2.76	2.08	3.67
	80–85	545	14%	114	20.9%	0.000	3.70	2.73	5.03
	> 85	790	21%	205	25.9%	0.000	4.91	3.72	6.47
Gender	Male	2136	55%	339	15.9%	0.226	1.12	0.93	1.33
	Female	1715	45%	248	14.5%	0.000	1.00	0.00	0.00
Albumin (g/dl)	3.5–4	374	10%	8	2.1%	0.000	1.00	0.00	0.00
	3–3.4	728	19%	39	5.4%	0.016	2.59	1.20	5.60
	2–3	2649	69%	303	14.2%	0.000	7.59	3.73	15.46
	< 2	520	14%	220	42.3%	0.000	33.55	16.30	69.05
BUN (mg/dl)	< 20	1690	44%	108	6.4%	0.000	1.00	0.00	0.00
	20–39	1479	38%	239	16.2%	0.000	2.82	2.22	3.59
	40–59	438	11%	130	29.7%	0.000	6.18	4.66	8.20
	≥60	244	6%	110	45.1%	0.000	12.03	8.74	16.54
RDW (%)	< 14.5	1647	43%	128	7.8%	0.000	1.00	0.00	0.00
	≥14.5	2204	57%	459	20.8%	0.000	3.12	2.54	3.84
Phosphorus (mg/dl)	1.51 < IP < 3.99	2841	74%	339	11.9%	0.000	1.00	0.00	0.00
	≤1.5	49	1%	14	28.6%	0.001	2.95	1.57	5.54
	4–4.49	479	12%	79	17.2%	0.002	1.53	1.16	2.01
	≥4.5	482	13%	155	32.2%	0.000	3.50	2.80	4.37
Magnesium (mg/dl)	1.35–2	3136	81%	406	12.9%	0.000	1.00	0.00	0.00
	≤1.35	342	9%	63	18.4%	0.005	1.52	1.13	2.03
	2–2.4	347	9%	105	30.3%	0.000	2.92	2.27	3.75
	≥2.4	26	1%	13	50%	0.000	6.72	3.10	14.61

Abbreviations: *OR* odds ratio, *CI* confidence interval, *BUN* blood urea nitrogen, *RDW* Red blood cell distribution width

**Fig. 1:**
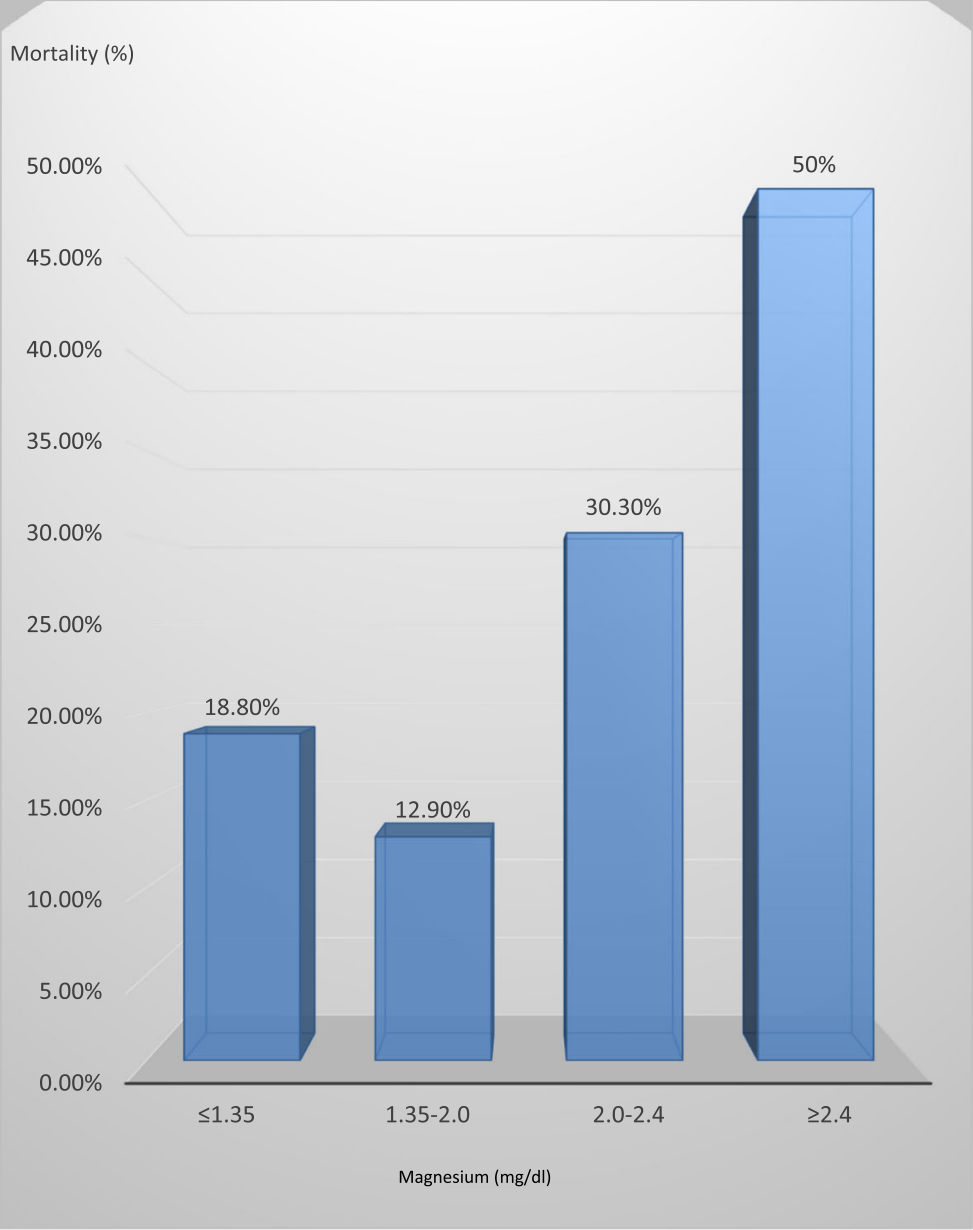
30 days mortality according to magnesium levels

Figure 1 demonstrates that hypomagnesemia and hypermagnesemia were associated with higher 30-day mortality rates (18.4%, OR 1.52, CI 1.13–2.03 and 50% OR 5.78, CI 2.66–12.53 respectively) compared to normomagnesemic group (14.8%). Thirty-day mortality rate was significantly higher (30.3%, OR 2.92, CI 2.27–3.75) in patients with magnesium levels within the upper normal limit (2–2.4 mg/dl) compared to the levels 1.35–2 mg/dl which has mortality rate of 12.9%.

### Relationship between phosphorous levels, magnesium levels and 30-day mortality

As shown in Table 2, abnormal magnesium levels were associated with high mortality rates regardless of all phosphorus levels.

**Table 2:** The association between the different phosphorus and magnesium levels with 30-day mortality

		Total		30 day mortality				95%CI	
		Number	Percent (%)	Number	Percent %	*P* value	OR	lower	Upper
Phosphorus (mg/dl)	Magnesium (mg/dl)	3851							
1.51–3.99	1.35–2	2370	62%	245	10.3	0.000	1.00	0.00	0.00
	≤1.35	256	7%	45	17.6	0.001	1.85	1.31	2.62
	≥2	215	6%	49	22.8	0.000	2.56	1.81	3.62
	Other	1010	26%	248	24.6	.	.	.	.
≤1.5	1.35–2	34	1%	9	26.5	0.726	1.00	0.00	0.00
	≤1.35	13	0%	5	38.5	0.424	1.74	0.45	6.71
	≥2	2	0%	0	0.0	0.999	0.00	0.00	0.00
	Other	3802	99%	573	15.1	.	.	.	.
≥4	1.35–2	698	18%	148	21.2	0.000	1.00	0.00	0.00
	≤1.35	68	2%	13	19.1	0.687	0.88	0.47	1.65
	≥2	153	4%	69	45.1	0.000	3.05	2.12	4.40
	Other	2932	76%	357	12.2	.	.	.	.
≥4.5	1.35–2	354	9%	101	28.5	0.002	1.00	0.00	0.00
	≤1.35	34	1%	9	26.5	0.799	0.90	0.41	2.00
	≥2	94	2%	45	47.9	0.000	2.30	1.44	3.67
	Other	3369	87%	432	12.8				

Abbreviations: OR-odds ratio, CI–confidence interval, IP-Inorganic Phosphorus, MG - Magnesium

### Relationship between blood urea nitrogen (BUN), magnesium, albumin, age and mortality

**Fig. 2:**
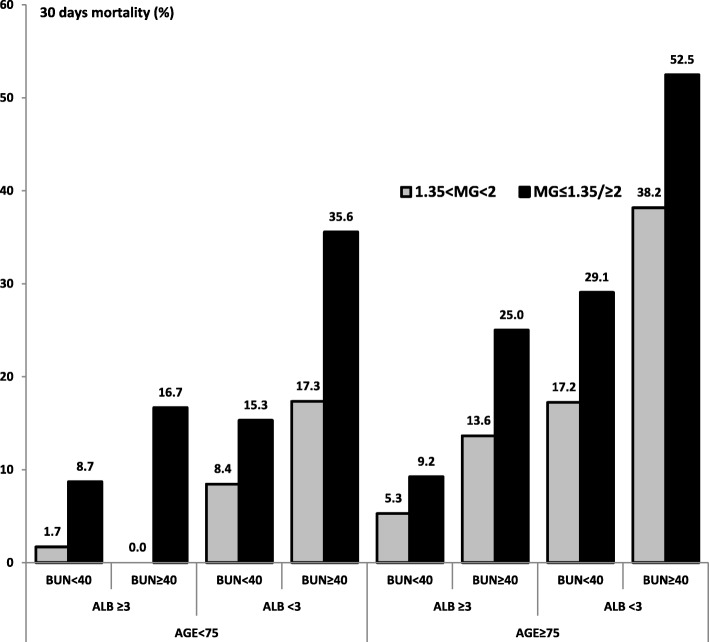
The relationship between magnesium, age, albumin, BUN and 30-day mortality

As shown in Fig. 2, the association between serum magnesium levels and 30-day mortality due to community acquired pneumonia outcome was maintained after adjustment for BUN, albumin and age.

### Multivariate analysis of factors associated with 30-day mortality

As shown in Table 3, in multivariate regression analysis, variables associated with increased risk of 30-day mortality include age > 75 years, BUN> 20 mg/dl, albumin < 3 g/dl, inorganic phosphorus levels> 4.5 mg/dl and abnormal levels of magnesium (hypermagnesemia, including high normal levels and hypomagnesemia).

**Table 3:** Multivariate analysis of parameters associated with 30-day mortality

Variables						Bootstrap for variable in equation					
		Coeff.	P	Adjusted	95% C.I.for OR		Bias	Std.	P	95% CI of B	
		B	value	OR	Lower	Upper		Error	value	Lower	Upper
Albumin (g/dl)	> 3		0.00								
	2–3	1.13	0.00	3.10	2.24	4.29	−0.03	0.18	0.010	0.83	1.53
	< 2	2.59	0.00	13.32	9.31	19.05	0.03	0.19	0.010	2.22	3.01
BUN (mg/dl)	≤20		0.00								
	20–39	0.57	0.00	1.77	1.37	2.29	0.02	0.14	0.010	0.28	0.92
	40–59	1.05	0.00	2.86	2.09	3.92	0.03	0.15	0.010	0.79	1.36
	≥60	1.28	0.00	3.61	2.49	5.24	0.01	0.20	0.010	0.97	1.72
Age (years)	≥75	0.93	0.00	2.53	1.98	3.22	−0.00	0.11	0.010	0.60	1.18
Magnesium (mg/dl)	≥2/≤1.35	0.63	0.00	1.87	1.42	2.48	−0.01	0.11	0.010	0.27	0.91
Inoraganic phosphorous (mg/dl)	≥4.5	0.83	0.00	2.29	1.77	2.96	0.00	0.11	0.010	0.58	1.18

Abbreviations: *OR* odds ratio, *CI* confidence interval, *BUN* blood urea nitrogen

In bootstrap multivariate analysis we show that all variables, including magnesium, which were significant in multivariate analysis were also significant in the model. The area under the curve for magnesium was 0.806 (95% CI 0.787–0.825).

## Discussion

In this retrospective study we studied the association between magnesium levels on admission and 30-day mortality in patients with community acquired pneumonia.

We found different 30-day mortality rates within the different magnesium levels: 18.8% in hypomagnesemia, 50% in hypermagnesemic, and 14.8% in normomagnesemic patients. This association was maintained even after adjustment for several parameters including albumin, BUN and age. Notably, magnesium levels within the upper normal limit, namely (2–2.4 mg/dl), were also associated with higher rates of 30-day mortality (30.3%).

Magnesium is an intracellular cation that possesses various physiological functions. It functions as a co-factor in intracellular enzymatic activities especially by chelating intracellular anionic ligands. Magnesium also competes with calcium in binding sites on proteins and membranes. It is involved in other cell cycle related processes including DNA and RNA synthesis, cell growth and reproduction, membrane structure, signal transduction modulation, and fat and protein synthesis [5, 13–15].

Magnesium deficiency is a common underdiagnosed problem in the ICU. Several studies have demonstrated high incidence of magnesium disturbances in ICU patients. Low Magnesium levels have been reported in approximately 50 % of ICU patients. The morbidity and mortality rates were significantly higher in these patients compared to patients with normal magnesium rates [16–20]. Rubeiz et al. [18] reported a 46% mortality rates in patients with hypomagnesemia in ICU. Conceivably, there were two-fold increase in mortality rates among patient with hypomagnesemia as compared to those with normal levels. Thus, the conclusion was that patients admitted with hypomagnesemia in ICU have an excess mortality rates.

It is interesting to assume that supplementation of magnesium to CAP may improve outcome. Therefore, it is conceivable to plan a prospective study to examine magnesium as a therapeutic modality in addition to the accepted treatment of CAP.

Hypermagnesemia, less frequently reported than hypomagnesemia, is found in 6–11% of patients admitted to ICU. It can also lead to cardiovascular and neuromuscular manifestations. The development of hypermagnesemia during the ICU stay is associated with higher morbidity and mortality rates and might be the direct result of prolonged disease, or sepsis [11, 21]. *Celi* et al. found that hypermagnesemic patients had a 2.5-fold greater likelihood of receiving intravenous vasopressors during the first 24 hours of ICU care, due to lower systemic blood pressure [22].*Guerin* et al. found that hypermagnesemia is associated with higher fatalities than hypomagnesemia [11]. Cheungpasitporn et al. found that respiratory diseases were associated with hypermagnesemia. This finding was probably attributed to magnesium mediated airway relaxation, immunomodulation and anti-inflammatory effects. Magnesium as an adjunctive therapy has been advocated for patients with moderate to severe airway diseases such as asthma despite inconclusive evidence of its benefit [23].

Magnesium is often given as a treatment of cardiac rhythm disturbances and status asthmaticus or for prevention of seizures and eclampsia [24, 25]. In the past, magnesium treatment was thought to be beneficial in treating ICU patients. This hypothesis was investigated by Broman et al. [13] who found that mild hypermagnesemia was associated with markedly worse survival parameters compared to normomagnesemic controls.

Our study has some limitations. First, is the retrospective nature of the study. It is important to emphasize that we did not have any influence on blood tests including magnesium levels which were taken according to the discretion of the treating physician. Secondly, our primary endpoint was all cause mortality. We did not have data regarding the specific cause of 30-day mortality. Third, data regarding that exact antibiotic agents prior to admission was missing in many patients. Therefore, we decided to exclude patients with prior antibiotic therapy. Fourth, data dealing with permanent medications were not available for many patients, with special emphasis on Proton Pump Inhibitors (PPI), a group of medication increasingly reported as a cause of hypomagnesemia. Fifth, the high mortality rates noticed in the study were due to selection bias since only hospitalized patients with pneumonia were included in the study.

## Conclusion

In conclusion, in this cohort study of patients with CAP, we demonstrated that abnormal magnesium levels on admission may be associated with increased 30-day mortality rates, compared to normal levels. Being a simple and readily available blood test, we believe that magnesium levels on admission in patients with CAP may play, in conjugation with other scores such as PASI or CURB65, a valuable role in stratifying these patients on the basis of 30-day mortality. Prospectively, well-designed, comprehensive studies are necessary to validate our findings before making any practical conclusions. Whether magnesium disturbances play a causative role in 30-day mortality in patients with CAP, or just being a surrogate marker, is a question to be answered by prospective interventional studies designed to examine to effect of correcting these disturbances on the final outcome.

## Abbreviations

AUC: Area Under Curve
BP: Systolic and diastolic blood pressure
BUN: Blood Urea Nitrogen
CAP: Community acquired pneumonia
CI: Confidence intervals
Hb: Hemoglobin
HR: Heart rate
Mg: Magnesium
OR: Odds ratios
pCO2: Partial pressure of carbon dioxide
PSI: Pneumonia Severity Index
RDW: Red blood cell distribution width
RHCC: Rambam Health Care Campus
RR: Respiratory rate
SPSS: Statistics Products Solutions Services
WBC: White blood cell count
